# Impact of Guideline-Directed Drug Therapy after ST-Elevation Myocardial Infarction on Outcome in Young Patients—Age and Sex-Specific Factors

**DOI:** 10.3390/jcm13133788

**Published:** 2024-06-27

**Authors:** Alicia Jeanette Fischer, Jannik Feld, Stefan A. Lange, Christian Günster, Patrik Dröge, Christiane Engelbertz, Thomas Ruhnke, Joachim Gerß, Holger Reinecke, Jeanette Köppe

**Affiliations:** 1 Department of Cardiology III—Adult Congenital and Valvular Heart Disease University Hospital Muenster, 48149 Muenster, Germany; 2 Institute of Biostatistics and Clinical Research, University of Muenster, 48149 Muenster, Germany; jannik.feld@ukmuenster.de (J.F.); joachim.gerss@ukmuenster.de (J.G.); jeanette.koeppe@ukmuenster.de (J.K.); 3 Department of Cardiology I—Coronary and Peripheral Vascular Disease, Heart Failure, University Hospital Muenster, 48149 Muenster, Germany; stefanandreas.lange@ukmuenster.de (S.A.L.); christianemaria.engelbertz@ukmuenster.de (C.E.); holger.reinecke@ukmuenster.de (H.R.); 4 AOK Research Institute (WIdO), 10178 Berlin, Germany; christian.guenster@wido.bv.aok.de (C.G.); patrik.droege@wido.bv.aok.de (P.D.); thomas.ruhnke@wido.bv.aok.de (T.R.)

**Keywords:** acute myocardial infarction, ST-segment elevation myocardial infarction, drug adherence, sex differences, determinants, mortality, health service research, real-world evidence

## Abstract

**Background**: Specifically young women are at risk for a poor outcome after ST-elevation myocardial infarction (STEMI). We aimed to investigate sex- and age-specific differences in outcome and associate these results with adherence to a guideline-directed optimal medical therapy (OMT). **Methods**: Administrative insurance data (≈26 million insured) were screened for patients aged 18–60 years with STEMI. Patient demographics, details on in-hospital treatment, adherence to OMT and its effect on mortality were assessed. Adherence to OMT was analyzed using multistate models and an association of those with death was fitted using multivariable Cox regression models with time-dependent co-variables. **Results**: Overall, 59,401 patients (19.3% women), median age 52 (interquartile range 48, 56) presented with STEMI. Female sex was associated with a poor outcome early after STEMI (90-day mortality: odds ratio 1.22, 95% confidence interval (CI) 1.12–1.32, *p* < 0.001). Overall survival was reduced in women compared to same-aged men. The ten-year survival rate was 19.7% (18.1–21.2%) versus 19.6% (18.9–20.4%) in men (*p* < 0.001). Although long-term drug adherence was low, its intake was associated with a better outcome. Specifically younger women showed a markedly lower mortality when on OMT (hazard ratio (HR) 0.22 (95% CI 0.19–0.26) versus HR 0.31 (95% CI 0.28–0.33) in men, *p*^int^ < 0.001). **Conclusions**: Specifically young women were at risk for a poor outcome in the early phase after STEMI. Although long-term adherence to OMT was low, it was generally associated with a lower mortality, specifically in women. Our findings emphasize on early and long-term preventive measures in all patients after STEMI.

## 1. Introduction

Patients who suffer ST-elevation myocardial infarction (STEMI) at a young age represent a relatively small proportion of the overall STEMI population [1]. Compared to older age groups, young patients are characterized by relevant differences in patient demographics, in-hospital treatment, and outcome [2]. While with advancing age, cardiovascular comorbidities are associated with a clustering of events, the number of cardiovascular comorbidities in younger patients is generally lower [1,3]. Apart from an influence of patient age on outcome after STEMI, previous data have shown profound sex-related differences [4,5,6,7]. For example, underlying mechanisms of myocardial infarction in young patients may differ from the most common mechanisms of infarction [8,9].

One key element of STEMI treatment consists of a stringent secondary prophylaxis [10]. In addition to lifestyle-altering measures, optimal medical therapy (OMT) in accordance with current guidelines is recommended and should be implemented early after the event [11,12,13]. In part, OMT targets the mitigation of severity and treatment of cardiovascular comorbidities [14,15]. Thus, the benefit of OMT in young patients with a lower number of cardiovascular comorbidities is less clear.

Although OMT is recommended to be initiated early after STEMI and be continued throughout the entire life of the respective patient, multiple analyses have shown that the compliance for OMT intake decreases with time [16,17]. Consistent drug intake depends on multiple factors such as socio-cultural differences, but there is also data indicating that age and sex play a role in adherence to OMT [7,18,19].

With this analysis, we aimed to assess healthcare-related data on adherence to OMT after STEMI in patients younger than 60 years. Data on the outcome after STEMI and the association between the outcome and OMT in the short- and long-term course after the event was analyzed. A focus was placed on sex-specific differences.

## 2. Materials and Methods

The analyzed data were based on the administrative dataset of the Federal Association of the Local Health Insurance Funds (Allgemeine Ortskrankenkasse, AOK). The dataset included unselected nationwide data of ≈26 million insurance holders (out of ≈83 million inhabitants in Germany). For reimbursement purposes, the health care provider is obligated to document all diagnoses and procedures in a standardized matter. In the dataset, all cardiac and extracardiac in- and out-of-hospital diagnoses are encoded using the German Modification of the International Statistical Classification of Diseases and Related Health Problems, 10th Revision (ICD-10-GM). Medical procedures are documented using the German Procedure Classification (OPS) codes. Drug prescriptions are encoded based on their respective Anatomical Therapeutic Chemical (ATC) codes. 

The health insurance database was screened over a study period of 10 years (2008 to 2018). Patients younger than 60 years with the in-hospital primary diagnosis of STEMI (index event) were selected for analysis. For inclusion, consistent data covering at least two years before the index event had to be present. If patients had inconsistent data concerning basic information, or patients entered the insurance company less than two years before the index event, they were excluded. To avoid double-counting of events, the primary in-hospital stay with diagnosis of STEMI was merged with the directly following in-hospital stays. A detailed flow-chart of the selection process and cohort definition is provided in Figure S1 (Supplementary Materials). 

Patients’ baseline characteristics, co-morbidities and details on in-hospital treatment were assessed based on in- and out-patient diagnostic and procedural codes. Data on OMT directly after the index event throughout a follow up of five years were assessed based on ATC drug codes (a full list of ICD-10-GM, OPS-procedure codes and ATC drug codes can be found in Table S1, Supplementary Materials). The number of classes of drugs prescribed was counted. According to current guideline recommendations, generally one of two antiplatelet drugs should be discontinued after one year [10]. To avoid miscounting due to a guideline-appropriate discontinuation of the second antiplatelet drug or additional intake of an oral anticoagulant (OAC), both antiplatelet drugs and/or OAC were counted as one. Thus, all included patients were expected to be discharged with cumulatively four different classes of drugs i.e., antiplatelet drugs and/or OAC, statins, beta blockers (BB) and ACE-inhibitors (ACEI), and Angiotensin II receptor blockers (ARB) after STEMI in this analysis. The intake of all four classes of drugs was defined as OMT. Predictors for 30- and 90-day mortality were assessed.

### 2.1. Missing Data 

With the exception of missing information about basic data, such as sex, date of birth or date of death (which were defined as exclusion criterions), there is no missing data in the study, since all variables were defined by existing ICD/OPS or ATC codes. If no related code was found, the variable was set to zero.

### 2.2. Endpoints

As endpoints, long-term survival restricted to patients who have survived at least 90 days after the event and overall survival (OS) were defined based on mortality information that were retrieved from the database. MACCE (major adverse cerebral and cardiovascular event), a composite endpoint including re-infarction, cerebral ischemia, resuscitation or death and re-infarction or death as individual parameters were defined as endpoints. The effect of guideline-directed medical therapy on long-term and OS was assessed. On the one hand, the effect of individual classes of drugs on the pre-defined end points was investigated, and on the other hand, the effect of the cumulative number of classes of drugs was assessed. 

### 2.3. Ethic Statement and Data Accessibility Statement

#### 2.3.1. Ethic Statement

In this analysis, only fully anonymized data was utilized. Consequently, no prior written informed consent had to be obtained. The study was conducted in accordance with the Declaration of Helsinki. This project was approved by the local Ethics Committee Westfalen-Lippe (No 2019-21-f-S). Informed consent was waived because this analysis is based on anonymized data.

#### 2.3.2. Data Accessibility Statement

The insurance data are protected by the German data protection laws (‘Bundesdatenschutzgesetz’, BDSG). As for data protection laws, the uncensored data cannot be made available in the manuscript, the supplemental files, or in a public repository. They are stored on a secure drive in the AOK Research Institute (WIdO) to facilitate replication of the results. Generally, access to data of statutory health insurance companies for research purposes is possible only under the conditions defined in German Social Law (SGB V § 287). Requests for data access can be sent as a formal proposal specifying the recipient and purpose of data transfer to the appropriate data protection agency. Access to the data used in this study can only be provided to external parties under the conditions of the cooperation contract of this research project and after written approval by the health insurance fund (wido@wido.bv.aok.de).

### 2.4. Statistical Analysis

The association of sex and short-term endpoints (30- and 90-day mortality) was analyzed using multivariable logistic regression models including age, sex, year of admission and patient’s comorbidity profile. OS was estimated using the Kaplan–Meier estimate. The association of sex on OS and long-term mortality (including only patients who survived the first 90 days after STEMI) was analyzed using multivariable Cox regression models with time-dependent co-variables. To account for different associations of OMT of OS and long-term mortality between the sexes, an interaction term sex × OMT was included in the models. OMT was represented in two different ways: first, including only the number of prescribed classes of drugs (0–4) and second, including each single drug in the models.

Moreover, the rate of patients with OMT during the course after STEMI was analyzed using multi-state models and the actual state probability was determined using Aalen–Johansen estimates. 

All analyses were fully exploratory (hypotheses generating), not confirmatory, and an adjustment for multiple testing was not performed. Statistical analyses were performed using SAS software V9.4, SAS Institute Inc., Cary, NC, USA and R version 4.1.0, R foundation, Vienna, Austria.

## 3. Results

### 3.1. Patient Characteristics

Overall, 59,401 adult patients younger than 60 years (median age 52, interquartile range (IQR) 48, 56) presented in-hospital with STEMI throughout the study period. Of those, 19.3% (*n* = 11,453) were women. All patient demographics divided in patients <60 years and a sub cohort of patients <45 years are displayed in Table 1.

As the focus of subsequent analyses was set on sex differences, data were additionally subdivided by sex. In all STEMI patients included in the analysis, coronary artery disease (CAD) had been documented in 38.3% (*n* = 22,737) before the index event. In the female cohort, 39.6% (*n* = 4531) presented with known CAD versus 38.0% (*n* = 18,206) of men. Of the patients <60 years included into analysis, 13.3% (*n* = 7927 patients) were younger than 45 years (median age 41 (IQR 38, 43)). Women accounted for 20.6% (*n* = 1635) of all patients <45 years. The overall number of patients with known CAD in patients <45 years was lower than in the cohort of patients <60 years (30.4% (*n* = 2406)). Other demographic parameters such as cardiovascular comorbidities (i.e., diabetes mellitus, arterial hypertension) were distributed variably with no clear association between patients’ age and sex. 

Patients of older age were more frequently under heart failure drug therapy at the time of STEMI, but there were no relevant differences between the sexes. Statins were generally more frequently taken by men (14.0% (*n* = 6712) versus 12.8% (*n* = 1462) in women). When focusing only on patients <45 years, the sex ratio remained constant with 7.7% (*n* = 487) of men versus 6.9% (*n* = 113) of women under statin intake. Classes of drugs prescribed for arterial hypertension, such as ACEI, ARB or BB, have generally been prescribed more frequently in women. Exemplary, 31.2% (*n* = 3574) of women and 27.4% (*n* = 13,144) of men were under ACEI or ARB. The number of patients <45 years taking antihypertensive drugs was lower, but the sex ratio remained constant with 18.4% (*n* = 300) of women and 16.3% (*n* = 1026) of men under ACEI or ARB therapy. Drug prescriptions prior to the event divided by age (<45 years and <60 years) and sex are displayed in Table 1.

Details on in-hospital treatment and complications are shown in Table 2. At the time of STEMI, 91.3% (*n* = 54,253) underwent PCI. Regarding patient sex, 89.8% (*n* = 10,282) of women and 91.7% (*n* = 43,972) of men received PCI. In the young cohort of patients <45 years, 91.0% (*n* = 7216) underwent a percutaneous coronary intervention (PCI). Of the women <45 years, 88.9% (*n* = 1454) received PCI, whilst men were treated with PCI in 91.6% (*n* = 5762).

### 3.2. Mortality and Predictors for 30- and 90-Day Mortality after STEMI

Within 30-days after STEMI, mortality was 6.0% (*n* = 3591) in all patients with relevant differences between the sexes (7.2% (*n* = 823) in women versus 5.8% (*n* = 2759) in men). The 90-day mortality was 6.6% (*n* = 3950) overall. Again, mortality was higher in women (7.9%, *n* = 898) than in men (6.4%, *n* = 3052). Mortality in patients <45 years was lower than in the overall cohort with the same sex ratio, respectively (Table 2). 

**Figure 1:**
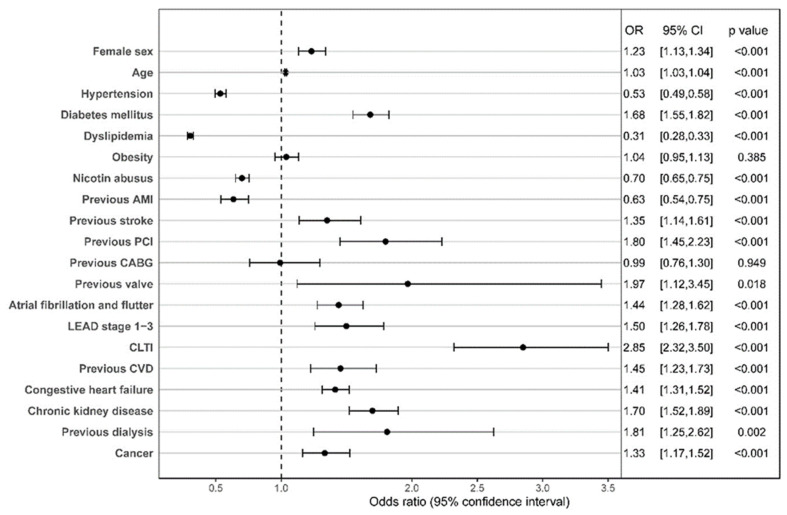
Multivariable logistic regression analysis of 30-day mortality. Risk factors for 30-day mortality based on the results of the multivariable logistic regression analysis in all patients < 60 years with in-hospital diagnosis of STEMI. AMI = acute myocardial infarction, PCI = percutaneous coronary intervention, CABG = coronary artery bypass graft, LEAD = lower extremity arterial disease, CLTI = chronic limb-threatening ischemia, and CVD = cardiovascular disease.

Figure 1 gives information on predictors for 30-day mortality. Multivariable logistic regression analysis for 90-day mortality can be found in the Supplementary Materials, Figure S2. A strong predictor for 30-day mortality was chronic limb threatening ischemia (CLTI). The odds ratio (OR) for 30-day mortality was 2.95 (95% CI (confidence interval) 1.21–3.50). Many cardiovascular comorbidities, such as diabetes mellitus and chronic kidney disease, were also associated with increased mortality. Patient sex had a profound impact on outcome, but its influence differed depending on the timing after the event. For 30- and 90-day mortality, female sex was associated with a reduced outcome with an OR of 1.23 (95% CI 1.13–1.34, *p* < 0.001) for 30-day mortality and an OR of 1.22 (95% CI 1.12–1.32, *p* < 0.001) for 90-day mortality.

**Figure 2:**
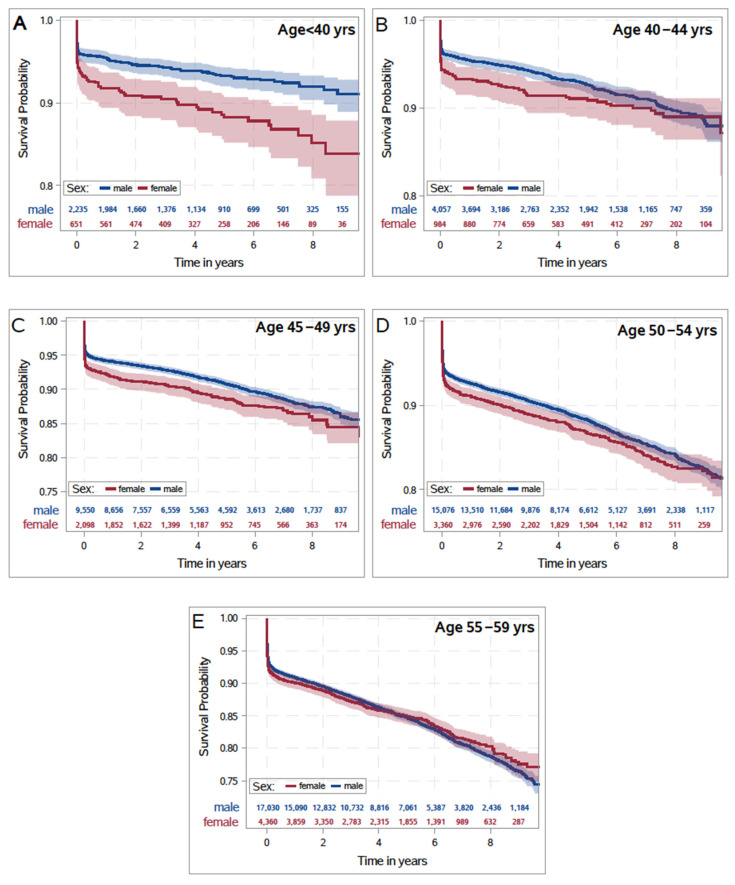
Survival probability according to age groups. Kaplan–Meier estimates for the overall survival probability after STEMI stratified by sex and age ((**A**) Age < 40, (**B**) 40–44, (**C**) 45–49, (**D**) 50–54, (**E**) 55–59).

Regarding OS, a poorer outcome in women compared to men was observed (*p* < 0.001). Accordingly, after one year, the overall OS rate was 7.6% (95% CI 7.4–7.9%). It was 9.0% (95% CI 8.4–9.5%) in women and 7.3% (95% CI 7.1–7.6%) in men. After 10 years, the survival rate was 19.6% (95% CI 18.9–20.3%) overall, with 19.7% (95% CI 18.1–21.2%) in women and 19.6% (95% CI 18.9–20.4%) in men (Table S2, Supplementary Materials). In patients <40 years, Kaplan–Meier estimates demonstrated a marked difference between women and men in survival probability, but no relevant differences in the patient cohort aged 55–59 years throughout a follow up of 10 years (Figure 2).

Event rates for MACCE (*p* = 0.061) and re-infarction or death (*p* = 0.195) were not notably associated with patient sex, however (Table S2, Supplementary Materials).

### 3.3. Clinical Effect of Guideline-Directed Drug Therapy on Mortality

**Figure 3:**
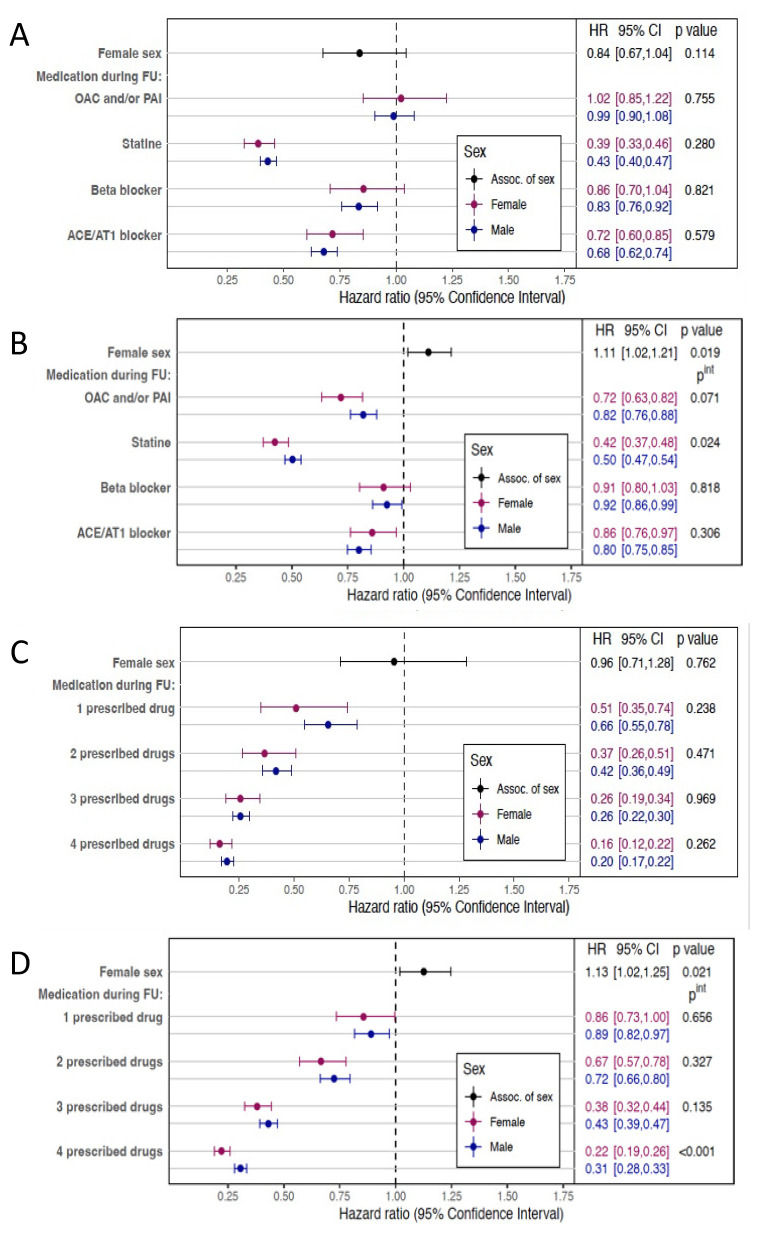
Forest plots of the different endpoints. (**A**) shows the effect of specific OMT drugs on long-term survival restricted to patients that survived the first 90 days after STEMI. (**B**) shows the effect of specific OMT drugs on overall survival (OS). (**C**) shows the effect of the number of classes of drugs taken on long-term survival restricted to patients that survived the first 90 days after STEMI. (**D**) shows the effect of the number of classes of drugs taken on OS.

The effect of a consistent medication intake on OS as well as long-term survival, restricted to patients who survived the first 90-days after the event, was assessed via Cox regression analyses (Figure 3). Statins, BB and ACEI/AT1 antagonists had a positive effect on long-term survival (Figure 3A) as well as on OS (Figure 3B) without statistically notable differences between men and women. Without relevant differences between the sexes, statins as an individual drug showed the most profound positive association with long-term survival (Hazard ratio (HR) 0.39 (95% CI 0.33–0.46) in women and HR 0.43 (95% CI 0.40–0.47) in men, *p*^int^ = 0.280). Statin intake was also associated with a general positive effect on OS. Relevant sex-related differences did not become overt (HR 0.42 (95% CI 0.37–0.48) in women versus HR 0.50 (95% CI 0.47–0.54) in men, *p*^int^ = 0.024). 

The outcome correlated positively to the number of classes of drugs taken, however. In the analysis on long-term survival, there were no relevant sex- differences (Figure 3C). There was an overall positive association between long-term survival and OMT with all four classes of drugs with a HR of 0.16 (95% CI 0.12–0.22) in women and a HR of 0.20 (95% CI 0.17–0.22) in men (*p*^int^ = 0.262). For OS, however, there were sex-specific differences (Figure 3D). OMT was associated with a lower mortality in all patients but specifically in women (HR 0.22 (95% CI 0.19–0.26) versus HR 0.31 (95% CI 0.26–0.33) in men, *p*^int^ < 0.001).

The HRs for patient sex are given as well as the interaction *p*-value comparing differences in outcome of women and men. All data are based on the results of the multivariable time-dependent Cox regression analysis for all patients < 60 years with diagnosis of STEMI stratified by sex.

Details on Cox regression analyses for overall survival can be found in Table S3 of the Supplementary Materials. Details on Cox regression analyses for long-term survival are documented in Table S4 of the Supplementary Materials. 

### 3.4. Guideline-Directed Drug Therapy after STEMI

**Figure 4:**
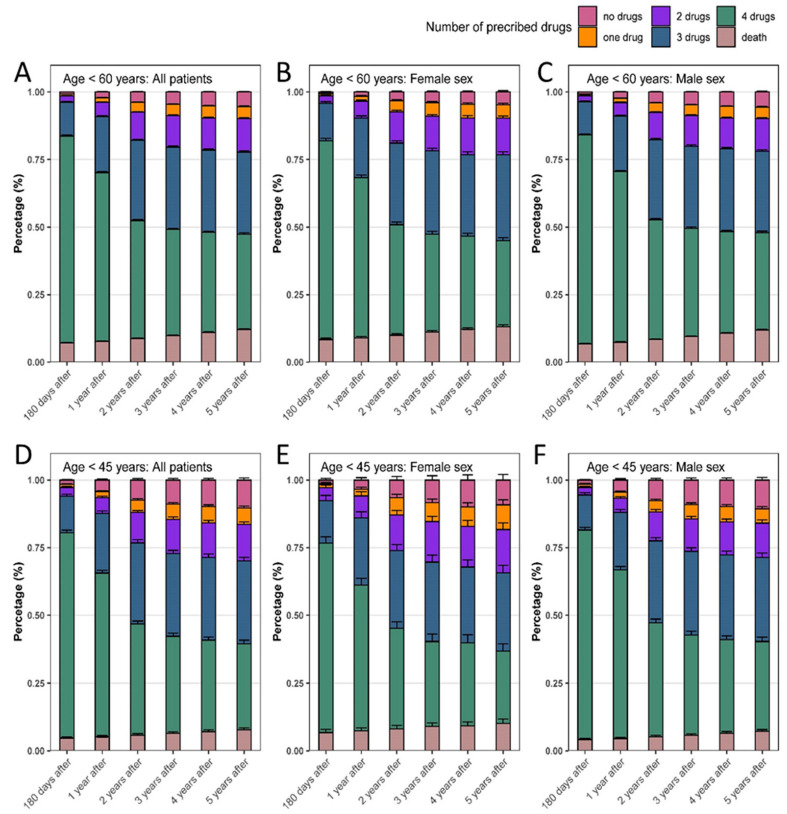
Prescription rates of guideline-directed drug therapy. Prescription rates covering a five-year period after STEMI in all patients < 60 years (**A**) as well as for women (**B**) and men (**C**) only. Separate analyses for STEMI patients < 45 years (**D**) generally as well as subdivided in women (**E**) and men (**F**) only.

Prescription rates of guideline-directed drug therapy were slightly different between men and women and varied between the age groups but generally decreased throughout the years. Figure 4 shows the prescription rates covering a five-year period after STEMI. 

Regardless of patient sex, there was a decrease in the frequency of drug prescriptions over time. Specifically, women < 45 years were affected by a low drug adherence. Within 180 days after STEMI, women < 45 years received prescription of an OMT in 74.9% of living patients, whilst after five years, only 29.6% were still under full therapy. In comparison, 80.7% of men took OMT within 180 days after STEMI and after five years, 35.8% of living patients were still under therapy. The highest guideline adherence five years after STEMI was seen in men < 60 with 40.9% (compared to 36.6% in women). The prescription rates subdivided by the respective drugs are noted in Table S5 of the Supplementary Materials.

## 4. Discussion

Overall, young patients showed a poor outcome after STEMI. Within the first 90 days after STEMI, young women were specifically at risk. This effect was drastic, as it led to a poorer overall mortality of young women compared to same-aged men. As one cornerstone of secondary prophylaxis, a guideline-directed optimal medical therapy is recommended, but adherence to drug therapy was generally low and decreased over time. Young women were identified as the cohort with the lowest drug adherence. By contrast, however, optimal medical drug therapy was positively associated with outcome in all STEMI patients with no relevant difference between the sexes. Our analysis showed that this positive effect did not relate to an individual substance, but its benefit increased gradually with the number of drugs from different classes taken. Intake of a full OMT was most beneficial for outcome. As all patients benefitted from OMT, the presented findings emphasize the importance of early and long-term drug treatment and patient education about relevance of drug adherence after STEMI irrespective of patient sex.

Of all patients identified in the analysis with STEMI, the majority of patients were male. This distribution between the sexes is consistent with other analyses [5,6,15]. Overall, in population-based studies, the median age of STEMI patients is approximately 65 years, and the sex ratio is 7:3 [6,15]. As women and young patients are underrepresented in non-selective analyses on STEMI, the herewith focus of our data may help in a more precise understanding of the specific patient cohort of young STEMI patients.

Cardiovascular comorbidities were less present in younger compared to older age groups, as it has been previously shown in comparable analyses [1,20]. Although patients suffered from fewer cardiovascular comorbidities, they had a marked effect on mortality as shown in the multivariable analyses. These results lead to the conclusion that cardiovascular comorbidities should be sought and treated to reduce morbidity in all STEMI patients irrespective of age and sex.

Overall mortality data gathered in this analysis were broadly consistent with previous analyses [21]. The generally poor outcome of young women compared to same-aged men has also been shown previously [22,23]. In first-time STEMI patients, we have shown previously that women were at specific risk early after the event [5]. The herewith presented data support this finding. Moreover, this analysis showed that the observation is generalizable to all young STEMI patients without focus on first-time or recurrent STEMI.

OMT is recommended as one cornerstone of secondary prophylaxis and the positive association between patient outcome and drug adherence has been shown previously [24,25]. One of the results of the herewith presented analysis was that the most positive effect on outcome was achieved, when all four drugs from different classes were taken with a gradual increase in benefit with the number of drugs taken. Thus, in clinical practice, focus should be given on a complete drug therapy irrespective of patient age or sex. Omission of one of the drug types will lead to a poorer outcome in young STEMI patients.

Of all individual drugs, statins had the most positive influence on outcome. Larsen et al. have shown previously that statin intake after STEMI is independently associated with less ischemic events as well as death [26]. Surprisingly, OAC and/or PAI individual drugs had no positive effect on outcome. Possibly, long-term bleeding complications, that have not been further investigated here, may have nullified the positive effects of this substance.

Although both sexes benefitted from the intake of secondary preventive drugs, small but significant differences between men and women only became overt, when all four classes of drugs were taken. Surprisingly, these sex-specific differences were only seen in the analysis that included patients who died within 90-days after STEMI. These differences between both sexes regarding outcome may be explained by biological differences between men and women, differences regarding their behavior such as motivation for drug adherence, but also the differences concerning the treatment by the responsible healthcare professionals: women are more frequently underdiagnosed in respect to their cardiovascular risk per se. Moreover, in the case of a cardiovascular event, women tend to be diagnosed and treated less frequently as well as with delay compared to men [27]. Without being able to conclusively state the reasons for this observation through this analysis, the result, however, stresses on the importance of early initiation of secondary preventive drug intake as it has been picked up in current guidelines [10]. Additionally, our results support the current guideline recommendation that no relevant differences should be made between men and women regarding secondary preventive drug therapy. 

One major concern in long-term treatment after STEMI is drug adherence. Underutilization of guideline-directed drug therapy after STEMI is a well-known problem [28]. Previous data assessed a treatment adherence of 66% over a median treatment period of 24 months [29]. We showed that drug adherence depended on timing after the event, patient sex, and age. For comparison, after 24 months, 40.3% of women and 44.4% of men <45 years were under OMT in this analysis. Intake decreased further throughout the years whilst generally, adherence was lower in women than in men. This observation appears paradox, as women benefit more from OMT than men regarding OS. 

Although the data showed sex-related differences, the general trend was equal and thus the consequences that should be taken of the given data are not sex-specific: Potentially, all young patients benefitted from consistent OMT. This therapy should be initiated early after STEMI and be continued long-term. The low adherence rate observed here indicates that long-term preventive measures should be followed to ensure optimal long-term care for these patients.

## 5. Strengths and Limitations

The presented analysis was based on a large dataset of unselected real-world data. Thus, by using these data, it is possible to examine subpopulations (e.g., young women) that are underrepresented in randomized controlled trials. Due to the mandatory reimbursement system in Germany, the data are characterized by high completeness and validity. About 30% of all hospital cases were reviewed regularly by independent physician task forces (Medizinischer Dienst der Krankenkassen) to ensure correct coding. However, coding errors and misclassifications may be present. The retrospective design and general constraints in the use of administrative care data have been described previously [30]. Only medications purchased by prescription from the corresponding patients were included in the analysis. Self-purchased medications without a prescription could not be included. The data on drug intake was based on drug prescription and is no definite proof that the prescribed drug has been taken. Parameters potentially of interest like the etiology of STEMI or clinical parameters such as hemodynamic values, laboratory findings, the ejection fraction or overall clinical morbidity cannot be accounted for due to the nature of administrative data. In general, the data were primary collected for financial purposes, not directly for research. 

## 6. Conclusions

Young patients are at risk for a poor outcome after STEMI. Specifically young women appear to be at risk in the early phase after the event. One key element of STEMI treatment is a secondary preventive drug treatment. Our data showed that adherence to an optimal medical therapy was low, especially in young women and decreased over time. However, all patients benefitted from optimal medical therapy irrespective of patient sex. Thus, our findings emphasize on the importance of early and long-term secondary preventive drug therapy in all patients after STEMI, with individualized consideration of the particular risk of the respective patient.

## Figures and Tables

**Table 1:** Patient demographics at the time of STEMI stratified by age ((**A**) <45 only and (**B**) <60 years) and sex.

(A)	Age < 45 Years		
	All	Women	Men
Patients—*n* (%)	7927	1635 (20.6)	6292 (79.4)
Median age—years (IQR)	41 (38, 43)	41 (37, 43)	41 (38, 43)
Number of involved coronary vessels—*n* (%)			
Unknown	769 (9.7)	189 (11.6)	580 (9.2)
1	4295 (54.2)	1023 (62.6)	3272 (52.0)
2	1696 (21.4)	270 (16.5)	1426 (22.7)
3	1167 (14.7)	153 (9.4)	1014 (16.1)
Arterial hypertension—*n* (%)	4759 (60.0)	981 (60.0)	3777 (60.0)
Atrial fibrillation and/or flutter—*n* (%)	247 (3.1)	51 (3.1)	196 (3.1)
Cancer—*n* (%)	219 (2.8)	62 (3.8)	157 (2.5)
Cerebrovascular disease—*n* (%)	95 (1.2)	24 (1.5)	71 (1.1)
CHF, all—*n* (%)	2134 (26.9)	460 (28.1)	1674 (26.6)
Chronic kidney disease—*n* (%)	406 (5.1)	108 (6.6)	298 (4.7)
Coronary artery disease—*n* (%)	2406 (30.4)	517 (31.6)	1899 (30.2)
Diabetes mellitus, unspecified—*n* (%)	1266 (16.0)	347 (21.2)	919 (14.6)
Dialysis	32 (0.4)	13 (0.8)	19 (0.3)
Dyslipidemia—*n* (%)	5172 (65.2)	980 (59.9)	4192 (66.6)
Ischemic cerebral insult—*n* (%)	100 (1.3)	33 (2.0)	67 (1.1)
Left ventricular congestive heart failure (CHF)—*n* (%)			
No CHF	5980 (79.4)	1208 (73.9)	4772 (75.8)
NYHA I	286 (3.6)	48 (2.9)	238 (3.8)
NYHA II	631 (8.0)	150 (9.2)	481 (7.6)
NYHA III	519 (6.5)	105 (6.4)	414 (6.6)
NYHA IV	511 (6.4)	124 (7.6)	387 (6.2)
Nicotine abuse—*n* (%)	3729 (47.8)	757 (46.3)	2972 (47.2)
Obesity—*n* (%)	2138 (27.0)	627 (38.4)	1511 (24.0)
Peripheral artery disease (PAD)—*n* (%)			
No PAD	7825 (98.7)	1607 (98.3)	6218 (98.8)
PAD 1–3	69 (0.9)	12 (0.7)	57 (0.9)
PAD 4–6	33 (0.4)	16 (1.0)	17 (0.3)
Previous acute myocardial infarction—*n* (%)	460 (5.8)	90 (5.5)	370 (5.9)
Previous coronary artery bypass graft—*n* (%)		***	***
Previous percutaneous coronary intervention—*n* (%)	129 (1.6)	22 (1.4)	107 (1.7)
Previous valve implantation—*n* (%)		***	***
Right ventricular heart failure—*n* (%)	166 (2.1)	41 (2.5)	125 (2.0)
ACE- inhibitors/ Angiotensin II receptor blockers—*n* (%)	1326 (16.7)	300 (18.4)	1026 (16.3)
Beta blockers—*n* (%)	970 (12.2)	258 (15.8)	712 (11.3)
OAC and/or PAI—*n* (%)	446 (5.6)	86 (5.3)	360 (5.7)
Oral anticoagulants (OAC)—*n* (%)	85 (1.1)	29 (1.8)	56 (0.9)
Platelet activation inhibitor (PAI)—*n* (%)	368 (4.6)	58 (3.6)	310 (4.9)
Statins—*n* (%)	600 (7.6)	113 (6.9)	487 (7.7)
All drugs—*n* (%)	159 (2.0)	27 (1.7)	132 (2.1)
**(B)**	**Total—Age < 60 Years**		
	**All**	**Women**	**Men**
Patients—*n* (%)	59,401	11,453 (19.3)	47,948 (80.7)
Median age—years (IQR)	52 (48, 56)	53 (48, 56)	52 (48, 56)
Number of involved coronary vessels—*n* (%)			
Unknown	4524 (7.6)	1098 (9.6)	3426 (7.2)
1	23,246 (39.1)	5327 (46.5)	17,919 (37.4)
2	16,271 (27.4)	2795 (24.4)	13,476 (28.1)
3	15,360 (25.9)	2233 (19.5)	13,127 (27.4)
Arterial hypertension—*n* (%)	43,474 (73.2)	8703 (76.0)	34,771 (72.5)
Atrial fibrillation and/or flutter—*n* (%)	3585 (6.0)	645 (5.6)	2940 (6.1)
Cancer—*n* (%)	3282 (5.5)	929 (8.1)	2353 (4.9)
Cerebrovascular disease—*n* (%)	2063 (3.5)	442 (3.9)	1611 (3.4)
CHF, all—*n* (%)	19,057 (32.1)	3679 (32.1)	15,378 (32.1)
Chronic kidney disease—*n* (%)	4776 (8.0)	1049 (9.2)	3727 (7.8)
Coronary artery disease—*n* (%)	22,737 (38.3)	4531 (39.6)	18,206 (38.0)
Diabetes mellitus, unspecified—*n* (%)	14,255 (24.0)	3086 (26.9)	11,169 (23.3)
Dialysis	201 (0.3)	56 (0.5)	145 (0.3)
Dyslipidemia—*n* (%)	42,413 (71.4)	7992 (69.8)	34,421 (71.8)
Ischemic cerebral insult—*n* (%)	1891 (3.2)	420 (3.7)	1471 (3.1)
Left ventricular congestive heart failure (CHF)—*n* (%)			
No CHF	42,310 (71.2)	8148 (71.1)	34,162 (71.3)
NYHA I	2157 (3.6)	356 (3.1)	1801 (3.8)
NYHA II	5038 (8.5)	959 (8.4)	4079 (8.5)
NYHA III	4706 (7.9)	881 (7.7)	3825 (8.0)
NYHA IV	5190 (8.7)	1109 (9.7)	4081 (8.5)
Nicotine abuse—*n* (%)	26,584 (44.8)	5264 (46.0)	21,320 (44.6)
Obesity—*n* (%)	15,250 (25.7)	3963 (34.6)	11,287 (23.5)
Peripheral artery disease (PAD)—*n* (%)			
No PAD	56,841 (95.7)	10,967 (95.8)	45,874 (95.7)
PAD 1–3	1825 (3.1)	328 (2.9)	1497 (3.1)
PAD 4–6	735 (1.2)	158 (1.4)	577 (1.2)
Previous acute myocardial infarction—*n* (%)	4449 (7.5)	812 (7.1)	3637 (7.6)
Previous coronary artery bypass graft—*n* (%)	976 (1.6)	132 (1.2)	844 (1.8)
Previous percutaneous coronary intervention—*n* (%)	1432 (2.4)	203 (1.8)	1229 (2.6)
Previous valve implantation—*n* (%)	105 (0.2)	23 (0.2)	82 (0.2)
Right ventricular heart failure—*n* (%)	1964 (3.3)	396 (3.5)	1568 (3.3)
	**Medication at Admission—*n* (%)**		
ACE- inhibitors/ Angiotensin II receptor blockers—*n* (%)	16,738 (28.1)	3574 (31.2)	13,144 (27.4)
Beta blockers—*n* (%)	12,327 (20.8)	3024 (26.4)	9303 (19.4)
OAC and/or PAI—*n* (%)	5608 (9.4)	946 (8.3)	4662 (9.7)
Oral anticoagulants (OAC)—*n* (%)	795 (1.3)	168 (1.5)	627 (1.3)
Platelet activation inhibitor (PAI)—*n* (%)	4937 (8.3)	805 (7.0)	4132 (8.6)
Statins—*n* (%)	8174 (13.8)	1462 (12.8)	6712 (14.0)
All drugs—*n* (%)	2196 (3.7)	312 (2.7)	1884 (3.9)

*** data < 10 censored due to privacy law.

**Table 2:** Details on in-hospital treatment at the time of STEMI stratified by age (<45 only and <60 years) and sex.

	Age < 45 Years			Total—Age < 60 Years		
	All	Women	Men	All	Women	Men
Mean length of hospital stay, years (±SD)	9.8 (±13.3)	11.1 (±17.3)	9.4 (±12.1)	10.2 (±13.2)	10.4 (±13.1)	10.2 (±13.2)
Mean costs, Euro (±SD)	8470.20 (±16,969)	9512.12 (±20,707)	8199.45 (±15,845)	9120.30 (±17,630)	8978.14 (±16,931)	9154.25 (±17,793)
Acute kidney failure and/or renal replacement therapy, n (%)	241 (3.0)	66 (4.0)	175 (2.8)	2475 (4.2)	494 (4.3)	1981 (4.1)
Acute kidney failure, n (%)	201 (2.5)	57 (3.5)	144 (2.3)	2083 (3.5)	406 (3.5)	1677 (3.5)
Bare metal stent only, n (%)	1178 (14.9)	221 (13.5)	957 (15.2)	8954 (15.1)	1562 (13.6)	7392 (15.4)
Bleeding, n (%)	441 (5.6)	107 (6.5)	334 (5.3)	3487 (5.9)	910 (8.0)	2577 (5.4)
Blood transfusion and/or bleeding, n (%)	702 (8.9)	209 (12.8)	493 (7.8)	6085 (10.5)	1618 (14.1)	4467 (9.3)
Blood transfusion, n (%)	362 (4.6)	137 (8.4)	225 (3.6)	3491 (5.9)	982 (8.6)	2509 (5.2)
Circulatory support device (Impella), n (%)	37 (0.5)	10 (0.6)	27 (0.4)	271 (0.5)	52 (0.5)	219 (0.5)
Coronary angiography, n (%)	7588 (95.7)	1555 (95.1)	6033 (95.9)	56,272 (95.2)	10,876 (95.0)	45,696 (95.3)
Coronary artery bypass grafting, n (%)	168 (2.1)	27 (1.7)	141 (2.2)	2192 (3.8)	320 (2.8)	1872 (3.9)
Drug eluting stent, n (%)	5605 (70.7)	1108 (67.8)	4497 (71.5)	42,625 (71.8)	8138 (71.1)	34,487 (71.9)
Extracorporeal membrane oxygenation, n (%)	95 (1.2)	20 (1.2)	75 (1.2)	760 (1.3)	159 (1.4)	601 (1.3)
GpIIb/IIIa-inhibitor, n (%)	2311 (29.2)	477 (29.2)	1834 (29.2)	16,374 (27.6)	2920 (25.5)	13,454 (28.1)
Hemorrhagic stroke, n (%)	***	***	***	144 (0.2)	36 (0.3)	89 (0.2)
Impella and/or intra-aortic balloon pump and/or extracorporeal membrane oxygenation or shock, n (%)	725 (9.1)	193 (11.8)	532 (8.5)	6423 (10.8)	1354 (11.8)	5069 (10.6)
In-hospital resuscitation, n (%)	591 (7.5)	161 (9.9)	430 (6.8)	4840 (8.1)	1063 (9.3)	3777 (7.9)
Intervention and/or thrombolysis, n (%)	7689 (97.0)	1583 (96.8)	6106 (97.0)	57,330 (96.5)	11,038 (96.4)	46,292 (96.6)
Intra-aortic balloon pump, n (%)	168 (2.1)	50 (3.1)	118 (1.9)	1475 (2.5)	291 (2.5)	1184 (2.5)
Percutaneous coronary intervention, n (%)	7216 (91.0)	1454 (88.9)	5762 (91.6)	54,253 (91.3)	10,282 (89.8)	43,971 (91.7)
Renal replacement therapy, n (%)	144 (1.8)	34 (2.1)	110 (1.8)	1312 (2.2)	257 (2.2)	1055 (2.2)
Sepsis, n (%)	111 (1.4)	30 (1.8)	81 (1.3)	1090 (1.8)	208 (1.8)	882 (1.8)
Shock, n (%)	663 (8.4)	175 (10.7)	488 (7.8)	5801 (9.8)	1230 (10.7)	4571 (9.5)
Stroke, n (%)	53 (0.7)	13 (0.8)	40 (0.6)	508 (0.2)	122 (1.1)	386 (0.8)
Thrombolysis, n (%)	145 (1.8)	35 (2.1)	110 (1.8)	904 (1.5)	195 (1.7)	709 (1.5)
Ventilation, n (%)	989 (12.5)	255 (15.6)	734 (11.7)	8564 (14.4)	1778 (15.5)	6786 (14.2)
Median duration—h (IQR)	77 (198)	69 (226)	81.5 (193)	72 (195)	57 (180)	76 (197)
In-hospital death, n (%)	336 (4.2)	101 (6.2)	235 (3.7)	3588 (6.0)	829 (7.2)	2759 (5.8)
30 days mortality, n (%)	331 (4.2)	94 (5.8)	237 (3.8)	3591 (6.0)	823 (7.2)	2768 (5.8)
90 days mortality, n (%)	357 (4.5)	103 (6.4)	254 (4.1)	3950 (6.6)	898 (7.9)	3052 (6.4)

*** data < 10 censored due to privacy law.

## Data Availability

The insurance data are protected by the German data protection laws (‘Bundesdatenschutzgesetz’, BDSG). As for data protection laws, the uncensored data cannot be made available in the manuscript, the Supplemental Files, or in a public repository. They are stored on a secure drive in the AOK Research Institute (WIdO) to facilitate replication of the results. Generally, access to data of statutory health insurance companies for research purposes is possible only under the conditions defined in German Social Law (SGB V § 287). Requests for data access can be sent as a formal proposal specifying the recipient and purpose of data transfer to the appropriate data protection agency. Access to the data used in this study can only be provided to external parties under the conditions of the cooperation contract of this research project and after written approval by the health insurance fund (wido@wido.bv.aok.de).

## Supplementary Materials

The following supporting information can be downloaded at: https://www.mdpi.com/article/10.3390/jcm13133788/s1, Figure S1: Study design; Figure S2: Multivariable logistic regression analysis of 90-day mortality; Table S1: ICD-10-GM Codes/ OPS diagnosis and procedure codes and ATC codes for medication relevant for analyses; Table S2: Event rates covering 10 years follow up of all STEMI patients < 60 years for the pre-defined endpoints overall survival, MACCE and re-infarction or death; Table S3: Cox-regression analysis for the endpoint overall survival; Table S4: Cox-regression analyses for the endpoint long-term mortality; Table S5: Relative prescription rates covering 6 months up to 5 years after STEMI.
