# Supplementary material for: Impact of Guideline-Directed Drug Therapy after ST-Elevation Myocardial Infarction on Outcome in Young Patients—Age and Sex-Specific Factors
Source: J Clin Med. 2024 Jun 27;13(13):3788. doi: 10.3390/jcm13133788 (PMC11242167; doi:10.3390/jcm13133788)
Supplement: Supplementary file 1 [file jcm-13-03788-s001.zip › jcm-2991738-supplementary.pdf]

Supplementary online only material

Figure S1: Study design.

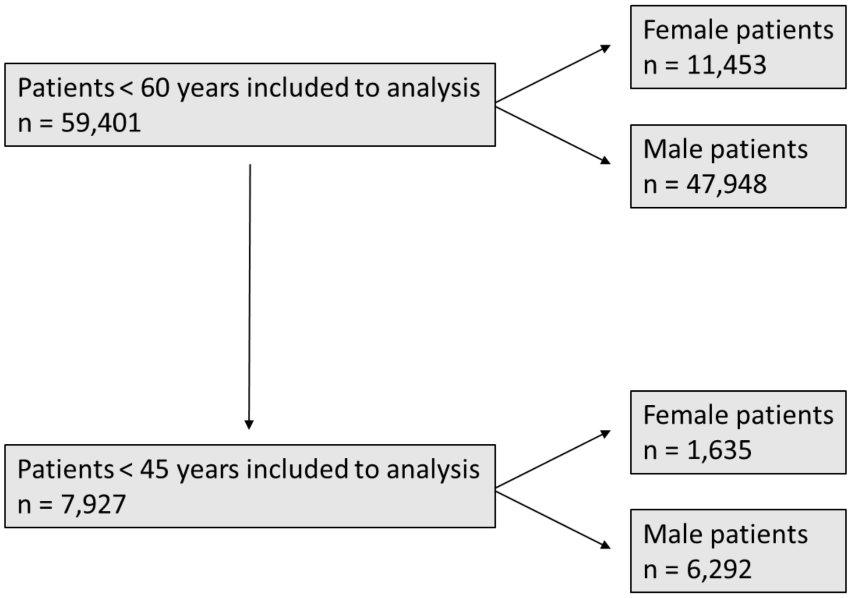

In total, n=59,401 patients younger than 60 years were included in the analysis, thereof n=11,453 female patients and n=47,948 male patients. Of all patients, 7,927 patients (n=1,635 female and n=6,292 male patients) were younger than 45 years.

Figure S2: Multivariable logistic regression analysis of 90-day mortality.

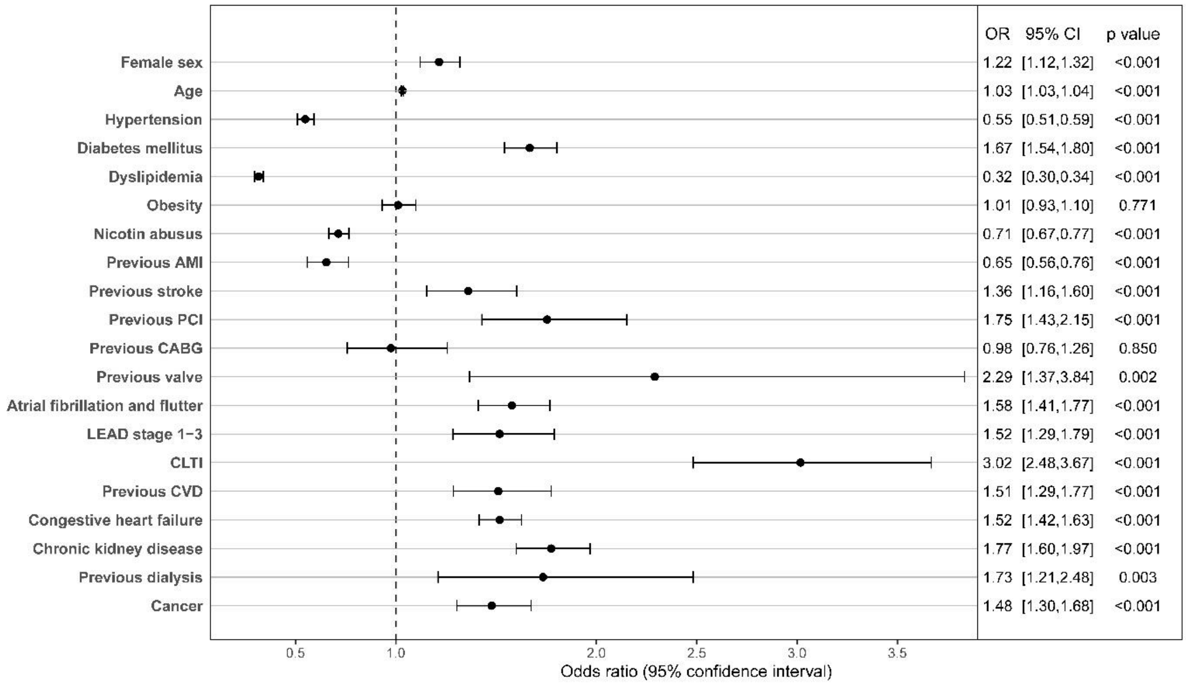

Risk factors of 90-day mortality based on the results of the multivariable logistic regression analysis in all patients < 60 years (n=59,401) with in-hospital diagnosis of STEMI.

AMI=acute myocardial infarction, PCI=Percutaneous coronary intervention, CABG=coronary artery bypass graft, LEAD=lower extremity arterial disease, CLTI=chronic limb-threatening ischemia, CVD=cerebrovascular disease.

Table S1: ICD-10-GM Codes/ OPS diagnosis and procedure codes and ATC codes for medication relevant for analyses.

Parameter	Codes
**ACE- inhibitors/ Angiotensin II receptor blockers**	ATC: C09A-D
**Acute kidney failure**	ICD: N17
**Acute myocardial infarction**	I21.-; I22.-
**Arterial hypertension**	ICD: I10-I15
**Atrial fibrillation and/or flutter**	ICD: I48
**Bare metal stent only**	OPS: 8-837.k
**Beta blockers**	ATC: C07
**Bleeding**	
**Blood transfusion**	OPS: 8-800.c
**Cancer**	ICD: C
**Cerebral insult, all**	ICD: I63, I64, I60, I61, I62
**Cerebrovascular disease**	ICD: I65, I66, I67.2
**CHF, all**	ICD: I50
**Chronic kidney disease**	ICD: N18, N19
**Circulatory support device (Impella)**	OPS: 8-83a.3
**Coronary angiography**	
**Coronary artery bypass grafting**	ICD: Z95.1, OPS: 5-36
**Coronary artery disease**	I25
**Diabetes mellitus, unspecified**	ICD: E10-E14
**Dialysis**	OPS: 8-853, 8-854, 8-855
**Drug eluting stent**	OPS: 8-837.m
**Dyslipidemia**	ICD: E78
**Extracorporeal membrane oxygenation**	OPS: 8-851, 8-852
**GpIIb/IIIa-inhibitor**	OPS: 6-002.j, 6-002.k, 6-002.n
**Hemorrhagic stroke**	ICD: K92, H44.8, T81.0, T81.2, T81.3, T81.7
**In-hospital resuscitation**	OPS: 8-771

**Intra-aortic balloon pump**	OPS: 8-83a.0
**Ischemic cerebral insult**	ICD: I63, I64, I69.3, I69.4
**Left ventricular congestive heart failure (CHF)**<br>**NYHA I**<br>**NYHA II**<br>**NYHA III**<br>**NYHA IV**	ICD:<br>I50.11<br>I50.12<br>I50.13<br>I50.14
**Nicotine abuse**	ICD: F17
**Number of involved coronary vessels**<br>**1**<br>**2**<br>**3**	ICD:<br>I25.11<br>I25.12<br>I25.13
**Obesity**	ICD: E66
**Oral anticoagulants (OAC)**	ATC: B01AA, B01AE07, B01AF (from 2013), B01AX06 (only 2012), B01AX08 (only 2012)
**Percutaneous coronary intervention**	OPS: 8-837
**Peripheral artery disease (PAD)**<br>**PAD 1-3**<br>**PAD 4-6**	ICD:<br>I70.20, I70.21, I70.22 (from 2015)<br>I70.22 (until 2014), I70.23, I70.24 I70.25 (from 2015)
**Platelet activation inhibitor (PAI)**	ATC: B01AC
**Right ventricular heart failure**	ICD: I50.0
**Sepsis**	ICD: A41
**Shock**	ICD: T81.1, R57.0
**ST- elevation myocardial infarction**	ICD: I21.0, I21.1, I21.2, I22.0, I22.1, I22.8
**Statins**	ATC: C10AA, C10BA, C10BX
**Thrombolysis**	OPS 8-020.8
**Valve implantation**	ICD: Z95.2, OPS: 5-351, 5-352, 5-353, 5-354; 5-535a

ACE =Angiotensin-converting-enzyme, CHF =congestive heart failure, OAC =oral anticoagulants, PAD = peripheral artery disease, PAI =platelet activation inhibitor

Table S2: Event rates covering 10 years follow-up of all STEMI patients < 60 years for the pre-defined endpoints overall survival, MACCE and re-infarction or death.

	**Total**	**Female sex**	**Male sex**	**P value**
**Overall survival**				<0.001
1 year rate	7.6% (7.4 – 7.9%)	9.0% (8.4 – 9.5%)	7.3% (7.1 – 7.6%)	
2 years rate	8.7% (8.4 – 8.9%)	9.9% (9.4 – 10.5%)	8.4% (8.1 – 8.6%)	
3 years rate	9.8% (9.5 – 10.0%)	11.2% (10.6 – 11.7%)	9.5% (9.2 – 9.7%)	
4 years rate	11.0% (10.7 – 11.2%)	12.2% (11.5 – 12.8%)	10.7% (10.4 – 11.0%)	
5 years rate	12.1% (11.9 – 12.4%)	13.2% (12.6 – 13.9%)	11.9% (11.6 – 12.2%)	
6 years rate	13.5% (13.2 – 13.9%)	14.3% (13.6 – 15.0%)	13.4% (13.0 – 13.7%)	
7 years rate	15.0% (14.7 – 15.4%)	15.7% (14.9 – 16.5%)	14.9% (14.5 – 15.2%)	
8 years rate	16.4% (16.0 – 16.8%)	16.9% (16.0 – 17.8%)	16.2% (15.8 – 16.7%)	
9 years rate	18.0% (17.6 – 18.5%)	18.4% (17.3 – 19.4%)	18.0% (17.4 – 18.5%)	
10 years rate	19.6% (18.9 – 20.3%)	19.7% (18.1 – 21.2%)	19.6% (18.9 – 20.4%)	
**MACCE**				0.061
1 year rate	16.0% (15.8 – 16.3%)	18.0% (17.3 – 18.7%)	15.6% (15.3 – 15.9%)	
2 years rate	19.5% (19.2 – 19.8%)	21.0% (20.3 – 21.8%)	19.1% (18.8 – 19.5%)	
3 years rate	22.4% (22.1 – 22.7%)	23.9% (23.1 – 24.7%)	22.1% (21.7 – 22.4%)	
4 years rate	25.0% (24.6 – 25.4%)	26.1% (25.2 – 26.9%)	24.8% (24.3 – 25.2%)	
5 years rate	27.6% (27.3 – 28.0%)	28.3% (27.4 – 29.2%)	27.5% (27.1 – 27.9%)	
6 years rate	30.4% (29.9 – 30.8%)	30.6% (29.6 – 31.6%)	30.3% (29.8 – 30.8%)	
7 years rate	33.0% (32.5 – 33.5%)	33.1% (32.0 – 34.1%)	33.0% (32.5 – 33.5%)	
8 years rate	35.4% (34.8 – 35.9%)	34.8% (33.6 – 35.9%)	35.5% (34.9 – 36.1%)	
9 years rate	38.1% (37.5 – 38.7%)	36.7% (35.4 – 38.0%)	38.5% (37.8 – 39.2%)	
10 years rate	40.3% (39.5 – 41.1%)	. . .	40.7% (39.8 – 41.5%)	
**Re-infarction or death**				0.195
1 year rate	14.9% (14.7 – 15.2%)	16.7% (16.0 – 17.4%)	14.5% (14.2 – 14.8%)	
2 years rate	18.0% (17.7 – 18.4%)	19.4% (18.7 – 20.2%)	17.7% (17.4 – 18.1%)	
3 years rate	20.6% (20.3 – 21.0%)	21.9% (21.1 – 22.7%)	20.4% (20.0 – 20.7%)	
4 years rate	23.0% (22.7 – 23.4%)	23.9% (23.1 – 24.8%)	22.8% (22.4 – 23.2%)	
5 years rate	25.4% (25.0 – 25.7%)	25.9% (25.0 – 26.8%)	25.2% (24.8 – 25.7%)	
6 years rate	27.9% (27.5 – 28.3%)	27.9% (27.0 – 28.9%)	27.9% (27.4 – 28.3%)	
7 years rate	30.3% (29.9 – 30.8%)	30.0% (29.0 – 31.1%)	30.4% (29.9 – 30.9%)	
8 years rate	32.4% (31.9 – 32.9%)	31.5% (30.4 – 32.6%)	32.6% (32.0 – 33.2%)	
9 years rate	35.0% (34.4 – 35.6%)	33.5% (32.3 – 34.7%)	35.4% (34.7 – 36.0%)	
10 years rate	37.1% (36.3 – 37.9%)	. . .	37.6% (36.8 – 38.5%)	

Event rates with 95% confidence intervals were given by cumulative incidence function determined using Aalen-Johansen estimates. Data are provided for all patients as well as women and men only. Differences between men and women were tested via two-sided log-rank test. Database contained n=59,401 patients younger than 60 years with STEMI.

Table S3: Cox-regression analysis for the endpoint overall survival.

Parameter	p value	Hazard ratio	95% Hazard ratio confidence limits	
**Age**	<.0001	1.030	1.026	1.034
**Arterial hypertension**	<.0001	0.768	0.727	0.810
**Atrial fibrillation and/or flutter**	<.0001	1.623	1.525	1.727
**Cancer**	<.0001	2.748	2.594	2.911
**Cerebrovascular disease**	<.0001	1.329	1.203	1.468
**Chronic kidney disease**	<.0001	1.609	1.515	1.709
**Diabetes mellitus, unspecified**	<.0001	1.470	1.397	1.546
**Dialysis**	<.0001	8.519	7.691	9.437
**Dyslipidemia**	<.0001	0.507	0.483	0.532
**Left ventricular congestive heart failure**	<.0001	1.552	1.481	1.626
**Nicotine abuse**	<.0001	0.910	0.870	0.953
**Obesity**	0.5293	1.018	0.964	1.074
**Peripheral artery disease 1-3**	<.0001	2.472	2.259	2.704
**Peripheral artery disease 4-6**	<.0001	1.386	1.272	1.511
**Previous acute myocardial infarction**	0.8519	0.992	0.909	1.082
**Previous coronary artery bypass graft**	0.9166	1.008	0.871	1.165
**Previous percutaneous coronary intervention**	<.0001	0.244	0.210	0.282
**Previous Stroke**	<.0001	1.417	1.288	1.560
**Previous valve implantation**	<.0001	2.457	1.853	3.257
**Sex**	0.0329	.	.	.
**Year of admission**	<.0001	0.978	0.969	0.988

Database contained n=59,401 patients younger than 60 years with STEMI. Multivariable Cox regression models including age, sex, year of admission and patient's comorbidity profile were used for analysis.

Table S4: Cox-regression analyses for the endpoint long-term mortality.

Parameter	p-value	Hazard ratio	95% Hazard ratio confidence limits	
**Age**	<.0001	1.029	1.022	1.035
**Year of admission**	<.0001	0.951	0.935	0.967
**Sex**	0.0656	.	.	.
**Arterial hypertension**	0.4372	1.034	0.950	1.125
**Dyslipidemia**	<.0001	0.774	0.72	0.833
**Obesity**	0.4048	0.968	0.897	1.045
**Nicotine abuse**	<.0001	1.145	1.073	1.222
**Previous acute myocardial infarction**	0.0226	1.135	1.018	1.265
**Previous Stroke**	<.0001	1.355	1.195	1.536
**Dialysis**	<.0001	8.073	7.214	9.033
**Previous percutaneous coronary intervention**	<.0001	0.224	0.188	0.266
**Previous coronary artery bypass graft**	0.6080	0.952	0.789	1.148
**Previous valve implantation**	0.0007	1.907	1.313	2.769
**Cerebrovascular disease**	0.0399	1.152	1.007	1.319
**Diabetes mellitus, unspecified**	<.0001	1.332	1.241	1.430
**Atrial fibrillation and/or flutter**	<.0001	1.502	1.376	1.640
**Peripheral artery disease 1-3**	<.0001	2.263	2.033	2.519
**Peripheral artery disease 4-6**	<.0001	1.264	1.138	1.405
**Cancer**	<.0001	3.893	3.627	4.178
**Chronic kidney disease**	<.0001	1.481	1.367	1.604
**Left ventricular congestive heart failure**	<.0001	1.679	1.570	1.796

Database contained n=55,451 patients younger than 60 years, who survived 90 days after the event of STEMI. Multivariable Cox regression models including age, sex, year of admission and patient's comorbidity profile were used for analysis.

Table S5: Relative prescription rates covering 6 months up to 5 years after STEMI.

Pharmaceutical Therapy	Time after STEMI	Age < 45 years		Total – Age < 60 years	
		Female	Male	Female	male
**OAC**	180 days	8.3 %	5.9 %	6.6 %	6.4 %
	1 year	5.1 %	3.6 %	4.9 %	4.8 %
	2 years	4.1 %	3.2 %	4.8 %	4.9 %
	3 years	4.5 %	3.3 %	5.2 %	5.3 %
	5 years	6.2 %	3.8 %	6.1 %	6.4 %
**PAI**	180 days	95.9 %	95.8 %	97.0 %	97.0 %
	1 year	93.4 %	92.3 %	94.5 %	93.7 %
	2 years	68.8 %	67.0 %	68.0 %	68.9 %
	3 years	60.8 %	62.4 %	62.5 %	64.4 %
	5 years	57.2 %	58.2 %	58.4 %	60.6 %
**OAC a./o. PAI**	180 days	96.6 %	96.3 %	97.7 %	97.5 %
	1 year	94.2 %	93.1 %	95.4 %	94.6 %
	2 years	70.4 %	68.5 %	70.5 %	71.8 %
	3 years	63.0 %	64.4 %	65.6 %	67.8 %
	5 years	60.5 %	60.7 %	62.6 %	64.7 %
**Statins**	180 days	92.1 %	93.6 %	94.8 %	95.5 %
	1 year	85.3 %	86.3 %	88.8 %	89.0 %
	2 years	80.0 %	82.6 %	84.6 %	85.5 %
	3 years	76.8 %	80.1 %	82.2 %	83.9 %
	5 years	73.9 %	78.7 %	80.5 %	82.5 %
**Beta blockers**	180 days	89.8 %	90.8 %	92.0 %	92.3 %
	1 year	82.9 %	83.0 %	85.5 %	85.3 %
	2 years	77.8 %	78.5 %	83.0 %	81.6 %
	3 years	76.5 %	75.5 %	81.4 %	80.0 %
	5 years	73.2 %	74.1 %	80.3 %	78.8 %
**ACE- inhibitors/ ARB**	180 days	83.7 %	89.7 %	89.0 %	91.4 %
	1 year	71.0 %	80.3 %	78.9 %	83.0 %
	2 years	67.7 %	75.5 %	76.1 %	80.1 %
	3 years	64.2 %	73.4 %	74.7 %	78.8 %
	5 years	62.0 %	72.7%	74.4 %	78.2 %
**All drugs**	180 days	74.9 %	80.7 %	80.3 %	82.9 %
	1 year	58.0 %	65.2 %	65.2 %	68.2 %
	2 years	40.3 %	44.4 %	45.5 %	48.4 %
	3 years	34.5 %	39.2 %	40.8 %	44.3 %
	5 years	29.6 %	35.8 %	36.6 %	40.9 %

The relative rates are related to living patients and divided into a cohort of patients < 60 years and < 45 years only as well as patients' sex. The database contained 59,401 patients aged < 60 years and 7,927 patients aged <45 years. The rate of patients with OMT during the course after STEMI was analysed using multi-state models and the actual state probability was determined using Aalen-Johansen estimates. Actual state probability was given relative to the living patients.
